# The Role of Leisure Activities as Markers of Cognitive Reserve on Dementia Risk in English Older Adults

**DOI:** 10.1093/geroni/igaa057.2297

**Published:** 2020-12-16

**Authors:** Pamela Almeida Meza, Andrew Steptoe, Dorina Cadar

**Affiliations:** University College London, London, England, United Kingdom

## Abstract

We examined the frequency of participation in cognitive and social type of leisure activities in association with dementia risk over 15 years of follow-up in 12,280 participants aged 50+ from the English Longitudinal Study of Ageing. Cox proportional hazards regression models were used to estimate the hazard of dementia in relation to the cognitive and social type of leisure activities as well as their interactions with sex and marital status. Medium and higher levels of engagement in cognitive leisure activities were associated with a lower risk of dementia. An analysis of the social type of leisure activities showed a similar pattern with protection for higher levels of engagement in a model adjusted for sex and marital status but further explained by wealth. This study shows a reduced risk of dementia for individuals with higher levels of engagement in cognitively stimulating activities, that may preserve cognitive reserve until later in life.

